# Shin Splint: A Review

**DOI:** 10.7759/cureus.33905

**Published:** 2023-01-18

**Authors:** Nikita Bhusari, Mitushi Deshmukh

**Affiliations:** 1 Musculoskeletal Physiotherapy, Ravi Nair Physiotherapy College, Datta Meghe Institute of Medical Sciences, Sawangi, Wardha, IND

**Keywords:** myofascial release (mfr) technique, overuse injury, athletes, pain, stretching

## Abstract

Medial tibial stress syndrome (MTSS), usually referred to as "shin splints," is a common overuse injury of the lower extremities affecting a large percentage of athletes. A variety of factors can lead to shin splints, including overtraining, poor footwear, muscular imbalances at the ankle, overtight or weak triceps surae muscles, imbalances at the thoracolumbar complex, and a body mass index (BMI) above 30. Injuries present with diffuse palpable pain that is often described as a dull ache following exercise. The pain is often alleviated by resting. Often, athletes complain of tenderness along the posteromedial edge of the tibia and pain along the middle to distal third of the posteromedial border of the tibia following an exercise session. The pain caused by a shin splint should be categorized according to its location and cause, such as lower medial tibial pain caused by periostitis or upper lateral tibial pain caused by raised compartment pressure. In order to prevent MTSS or shin splints, it is important to avoid excessive stress. The main objectives of shin splint treatment are to relieve pain and to enable the patient to return to normal activities without pain. To prevent shin splints, repetitive stress should be avoided. In this paper, we review what is known about the pathophysiology of shin splint syndrome, present evidence regarding risk factors associated with shin splints, assess the effectiveness of prevention strategies, and make recommendations for prevention. The purpose of this study is to assess the effectiveness of interventions to prevent shin splints.

## Introduction and background

Pain along the shinbone (tibia) is termed "shin splints," which occurs due to the inflammation of the tissue in the area. It is also known as medial tibial stress syndrome (MTSS). Military recruits, runners, and dancers are all at risk of shin splint. Athletes who have recently increased or modified their training programs are prone to this, as well as athletes who have not properly warmed up, increased their training mileage suddenly, or have hyperpronated their feet. Muscles, tendons, and bone tissues are overworked as a result of increased exercise. For an athlete, this kind of injury can disturb their performance and all their activities [1]. A stress fracture of the tibia, which is represented by focused discomfort in the front tibia, is the most frequent complication of medial tibial stress syndrome [2]. The discomfort gradually lasts longer into the run as a patient's injury gets worse; eventually, it lasts through the cooldown and into daily activities and can localize to become point soreness [3]. People with a higher body mass index (BMI) and those who had previously used orthotic devices for a long time were shown to be more likely to develop diabetes. This condition affects the lower leg [4].

The most frequent overuse symptoms are medial tibial stress syndrome, pains in the knee extension mechanism, Achilles tendon peritendinitis, iliotibial tract friction syndrome, retrocalcaneal bursitis, discomfort in the metatarsal arch, stress fractures of the tibia, plantar fasciitis, Osgood-Schlatter's disease, and chronic calf muscle pains [5]. Medical history and physical examination are frequently used to diagnose shin splints. An X-ray or other investigations such as magnetic resonance imaging or a bone scan can sometimes help detect other potential causes of pain, such as a stress fracture. Physiotherapy and orthotics are frequently used not just to prevent medial tibial stress syndrome but also to treat it. Rest often relieves the pain of shin splints; without any break in training, the pain will grow more and also serious. To rule out the possibility of medial tibial stress syndrome in an athlete, several factors should be addressed [6]. The majority of athletes who suffer from shin splints never have any severe performance limitations, never visit a doctor, and can continue training without experiencing any running-related limitations [7]. Some medical professionals think that any pain in the shins brought on by effort should be referred to as "shin splints." Others contend that the term should only be applied to a limited number of clinical entities [8]. Female athletes are more prone to medial tibial stress syndrome than male athletes, according to studies [6].

According to one theory, medial tibial stress syndrome puts a load on the skeleton when there is a lot of activity. The fascia along the tibia's medial border might be released, allowing easy access to the tibia and adjacent soft tissues while also relieving the strain on the periosteum [9]. The design and methodological quality of surgical outcome studies done on medial tibial stress syndrome are not so good. The most successful surgical methods involve a deep posterior compartment, which includes a soleus sling and a strip of posteromedial tibia periosteum. People having vitamin D deficiency, osteoporosis, and flat feet have a higher chance of getting shin splint [10]. In long-term situations, plain radiographs and non-contrast CT (NCCT) scan can reveal focal increased cortical thickening or exostosis. The bone scan, which is used to detect early indications, has been superseded by MRI. The connecting soft tissue structures are best identified in this three-dimensional research. The severity of the problem can also be determined by the surrounding periosteal edema. This is by the tibial circumference being covered. Mild will be defined as 50% of the total. Almost always, the disease's progression is self-contained. Ice packs, nonsteroidal anti-inflammatory drugs (NSAIDs), and some degree of rest should be part of the regimen [11]. People with MTSS frequently have bilateral soreness or pain in the medial side of the tibia, most frequently in the distal region. The medial tibia's middle part is a typical location for pain. Shin splints, on the other hand, can affect the full length of the leg [8]. Lateral side pain is frequently described as dull and unpleasant. Pain is more frequently felt at the beginning of a workout and fades as the workout progresses. It increases with movement and decreases with rest [12]. The discomfort normally gets worse the next day, but it will eventually go away. In cases of severe and ongoing medial tibial stress syndrome, pain may be experienced even while the patient is at rest. Both dysesthesia and radiation to the foot have been noted. Similar signs and symptoms are seen in both stress fractures and other overuse injuries [13]. Therefore, it is crucial to keep a keen sense of suspicion.

Other characteristics of medial tibial stress syndrome include pain during percussion and discomfort during hopping; it has been shown that the posteromedial border of the tibia is the most sensitive area [14]. Patients with this condition may also experience mild edema and tibial line subcutaneous thickening. It is critical to distinguish this from a callus that can be seen. During the examination, passive stretching may reveal weakness and pain. Localized pain, discomfort, and edema are more likely to be brought on by stress fractures. As a result, they need to be medically excluded. Additionally, conditions such as tendinitis, compartment syndrome, popliteal artery compression, vasculitis, and nerve impingement are taken into account when evaluating people with medial tibial stress syndrome.

**Figure 1:**
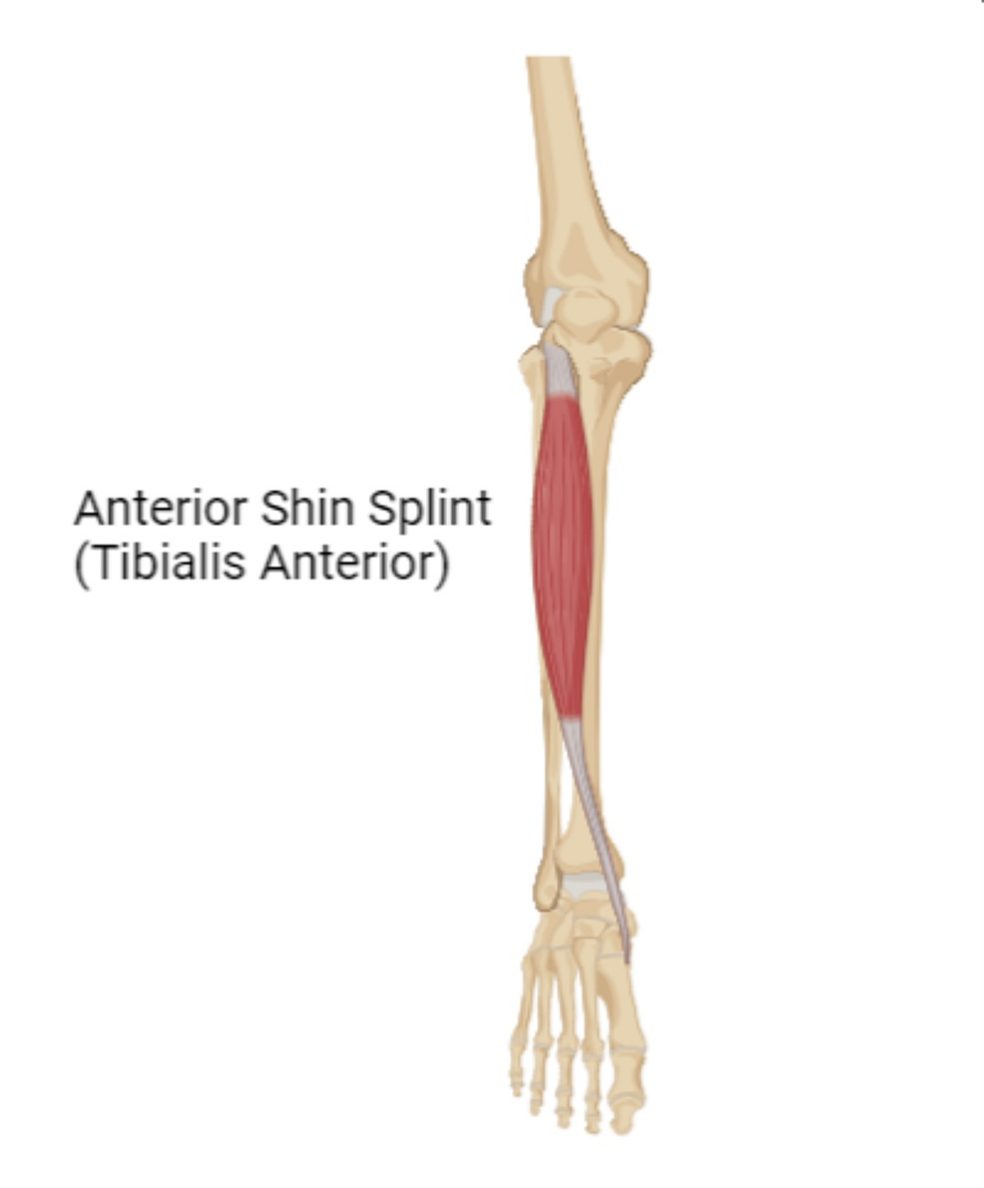
Anterior shin splint.

This review article reviews the effective treatments for MTSS that reduce pain and improve functions within a short period of time, allowing individuals to continue their normal activities. Clinical practitioners will benefit from this article as it provides a brief overview of the condition that would be useful for future studies in the same condition (Figure 1).

## Review

Methodology

**Figure 2:**
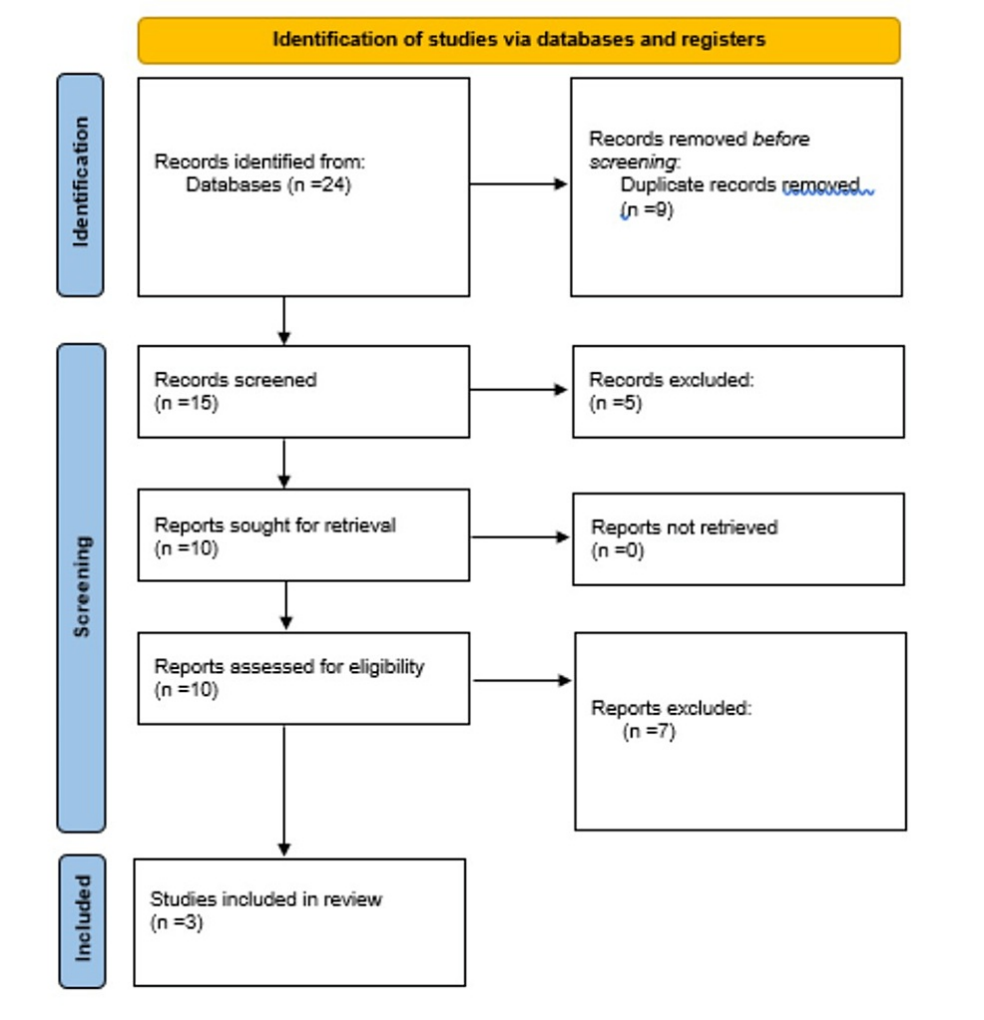
PRISMA flow chart. PRISMA: Preferred Reporting Items for Systematic Reviews and Meta-Analyses

The keywords medial tibial stress syndrome, athletes, tibialis anterior, and shinbone were used to search databases for relevant peer-reviewed articles. In this study, relevant articles were screened and included. Research papers, original articles, systematic reviews, literature reviews, case-control studies, randomized trials, and cross-sectional studies were considered. By evaluating and categorizing inclusion and exclusion criteria, the review article examines the influence of flexibility exercises, compression bandage, supportive insoles, and rest on relief from shin splint (Figure 2).

Causes

**Figure 3:**
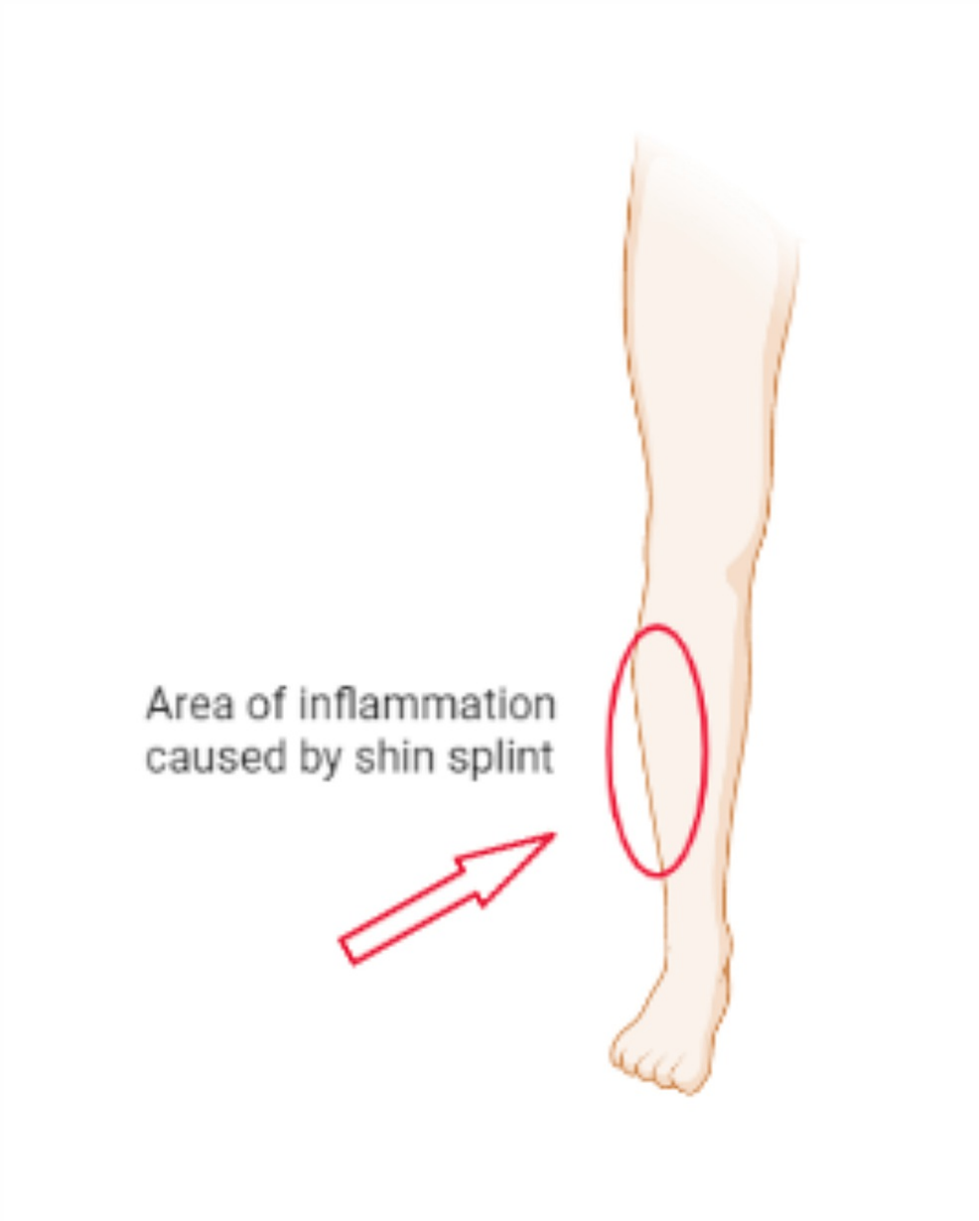
Area of inflammation.

Shin splints are brought on by persistent strain on the connective tissues that attach your muscles to the bone and the shinbone [15]. Shin splints typically occur as a result of overuse injuries to the leg's muscle and bone tissue (periosteum). Usually, shin splints appear after abrupt changes in physical activity. These may entail frequency modifications, such as increasing the number of days you work out each week. Shin splints can also develop as a result of length and intensity increases, such as jogging uphill or for longer distances. Shin splints can also result from having flat feet or unusually inflexible arches, as well as from exercising in unsuitable or worn-out footwear. The majority of people who get shin splints are runners. Military recruits and dancers are two additional categories who regularly receive the diagnosis (Figure 3) [16].

Treatment

Nonsurgical Treatment

Rest: Since shin splints are often brought on by overuse, the standard course of treatment includes taking several weeks off from the painful activity. During your rehabilitation, substitute lower-impact cardio exercises such as swimming, riding a stationary bike, or utilizing an elliptical trainer.

Nonsteroidal anti-inflammatory medicines: Pain and swelling are lessened by medications including ibuprofen, aspirin, and naproxen.

Ice: Several times a day, apply ice packs for 20 minutes at a time. Do not immediately apply ice to the skin.

Compression: A compression bandage made of elastic may stop further swelling.

Flexibility exercises: Your shins may feel better after stretching the muscles in your lower legs.

Supportive shoes: During regular activities, wearing shoes with good cushioning will assist in lessening stress on your shins.

Orthotics: Orthotics may be helpful for those with flat feet or persistent shin splint issues [17]. By correcting and supporting your foot and ankle with shoe inserts, you can lessen the strain on your lower leg [18]. You have the option of having orthotics made particularly for your foot or purchasing them "off the shelf."

Supportive insoles: It ensures that your feet and legs are operating in the right alignment and aids in the treatment of shin splints. This helps to avoid problems that put too much strain on the muscles that stabilize your ankles, allowing your shin splints to recover [19].

Return to exercise: Rest and the simple treatments listed above usually help shin splints go away. You should be pain-free for at least two weeks before beginning an exercise regimen once more. Keep in mind that you must exercise more gently when you start up again. Exercise should not be done as frequently or for the same amount of time as it was in the past. Make sure you stretch and warm up appropriately before exercising. Gradually, increase your exercise. As soon as you feel the same pain, stop exercising. Take a day or two off of work, and use a cold pack. Resuming your workouts at a lower intensity is a good idea. Train harder but more gradually than before.

Surgical Treatment

Surgery is only occasionally necessary for shin splints. In cases that are extremely severe and if nonsurgical treatment is ineffective, surgery has been performed. However, the effectiveness of surgery is unclear [20].

Review of literature

In 2015, Cheatham et al. assessed and critically evaluated current data to answer the following questions: Can self-myofascial release (SMR) using a foam roll or roller massager improve the range of motion (ROM) without affecting muscle function? Is self-myofascial release using a foam roller or roller massager effective for improving postexercise muscle repair and reducing delayed-onset muscle soreness (DOMS)? Does self-myofascial release using a foam roller or roller massager before an activity improve muscle performance? A search strategy was implemented prior to April 2015 that incorporated electronic databases, as well as well-known journals. This study included studies that met the following criteria: 1) peer-reviewed articles published in the English language; 2) several studies that have examined the effects of self-myofascial release (SMR) using a foam roll or roller massager on joint range of motion, acute muscle soreness, and delayed-onset muscle soreness (DOMS); 3) studies comparing a foam roll or roller massager intervention program to a control group; and 4) the comparison of two foam roll or roller massager intervention approaches. The PEDro scale was used to evaluate the papers' quality. Fourteen articles met the requirements for inclusion. With the use of a foam roller or roller massager, self-myofascial release tends to increase joint range of motion without sacrificing muscle performance in the near term and may impact muscle performance and delayed-onset muscle soreness after intense exercise. Short bouts of self-myofascial release before exercise do not appear to affect muscle performance. The body of research on the effects of SMR is still in its infancy. This study suggests that foam rolling and roller massage may be effective therapies for improving joint range of motion and muscle performance both before and after exercise. Despite this, because different studies use different approaches, there is currently no consensus on the best SMR program [1].

In 2002, in their study, Couture and Karlson said that experts are divided over the origin of MTSS. When the cause is unknown, prevention can be challenging. Increased foot pronation, stronger plantar flexor muscles, a sudden increase in training volume, a low calcium intake, a hard or sloped (or both) running surface, the wrong footwear, and a history of injury are all thought to be risk factors for MTSS. It is near impossible to control all these risk factors for all our athletes unless we fully understand the true causes of shin splints [15].

In 2013, according to Mendiguchia et al., one of the most typical lower leg injuries in sports is medial tibial stress syndrome. According to a high-quality appraisal of the literature on MTSS prevention, it accounts for 6%-16% of all running injuries and up to 50% of all lower leg injuries. The following have been proposed as risk factors for MTSS: increased foot pronation, strengthened plantar flexors, increased forefoot or hindfoot (or both) varus tendency, abruptly increased training volume, inadequate calcium intake, hard or inclined running surfaces, poor footwear, and prior injury [8].

Incidence

**Figure 4:**
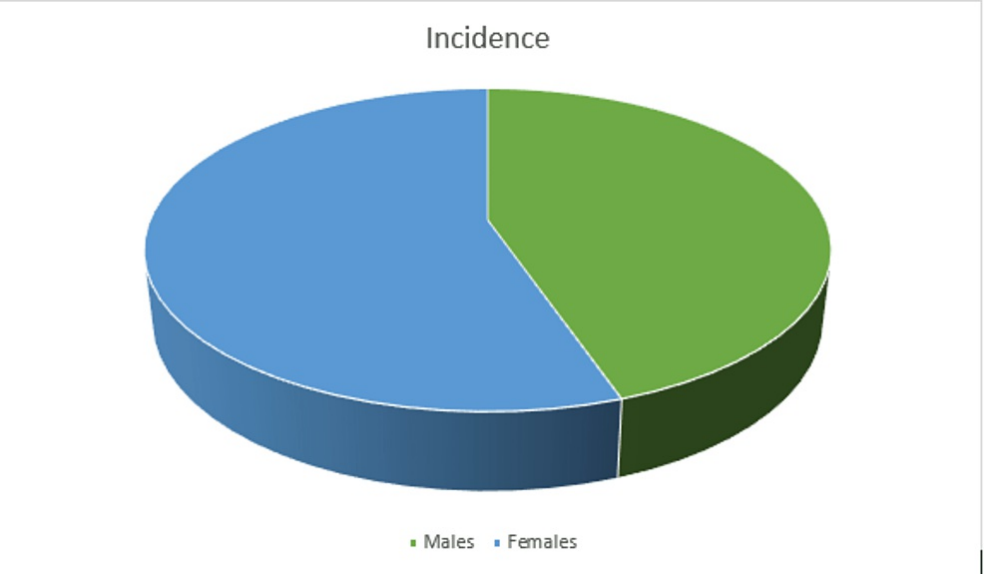
Pie chart representing incidence among males and females.

Males (44.7% of the population) were less likely to have a shin splint than females (55.3%) (Figure 4). Depending on the degree of pain and the shoe surface, shin splints are more frequent in marathon runners [21].

Discussion

One of the most frequent injuries suffered by athletes is medial tibial stress syndrome (MTSS). Affected individuals may be more susceptible to such injuries due to biomechanical anomalies. It is an early stress injury in the progression of tibial stress fractures and manifests as exercise-induced pain over the anterior tibia. Significant causative factors include training errors as well. A significant increase in workload, volume, and high-impact exercise can put people at risk of MTSS. Rest and recovery should be stressed as key components of sports training because shin splints are caused by a mechanical overload of various components of the leg's musculoskeletal system that exceeds their capacity for adaptive reconstruction. The severity and length of the damage are decreased by a timely and accurate diagnosis. Physical therapists improve the quality of life through hands-on care, patient education, and prescribed movement. The goal of treatment should be to decrease discomfort and inflammation while also identifying any potential biomechanical issues that might be resolved with stretching and strengthening routines or by using an orthotic device. The best kind of treatment is prevention since it stops an injury from getting worse.

## Conclusions

There are a variety of patterns of sports injuries with various types and natures of sports events, but shin splint is one of the most common. Lower leg pain is the most common symptom of shin splints. Depending on the severity of the pain, the shin bone may feel tender to the touch. MTSS is a common injury among athletes who seek treatment at sports injury centers. Therefore, sports persons should be more aware of the risk factors of shin splints and should be aware of how to treat and prevent them. A variety of clinical investigations can be conducted to determine the condition. CT and MRI scans can reveal the exact location of the condition. This article has discussed various interventions that can be used to manage shin discomfort and ankle dorsiflexion range of motion in participants with medial tibial stress syndrome. This article offers a brief overview of the condition to aid clinical practitioners, which will be useful for future studies on the same issue.
